# Effect of Electroacupuncture on Hyperalgesia and Vasoactive Neurotransmitters in a Rat Model of Conscious Recurrent Migraine

**DOI:** 10.1155/2019/9512875

**Published:** 2019-05-14

**Authors:** Xiaobai Xu, Lu Liu, Luopeng Zhao, Bin Li, Xianghong Jing, Zhengyang Qu, Yupu Zhu, Yajie Zhang, Zhijuan Li, Marc Fisher, Brain E. Cairns, Linpeng Wang

**Affiliations:** ^1^Acupuncture and Moxibustion Department, Beijing Hospital of Traditional Chinese Medicine, Capital Medical University, Beijing Key Laboratory of Acupuncture Neuromodulation, Beijing, China; ^2^Beijing Hospital of Traditional Chinese Medicine, Capital Medical University, Beijing Institute of Traditional Chinese Medicine, Beijing, China; ^3^Institute of Acupuncture and Moxibustion, China Academy of Chinese Medical Sciences, Beijing, China; ^4^Division of Stroke and Cerebrovascular Diseases, Department of Neurology, Beth Israel Deaconess Medical Center, Boston, MA, USA; ^5^Faculty of Pharmaceutical Sciences, The University of British Columbia, Vancouver, Canada; ^6^Center for Neuroplasticity and Pain, SMI, Department of Health Science and Technology, The Faculty of Medicine, Aalborg University, Aalborg E, Denmark

## Abstract

Migraine onset is associated with the abnormal release of vasoactive neurotransmitters from perivascular nerves, and these neurotransmitters are involved in the pathophysiology of migraine. Hyperalgesia is a key feature of migraine, and accumulating evidence indicates that electroacupuncture (EA) at the single acupuncture point (Fengchi [GB20]) is effective in ameliorating hyperalgesia. In clinical practice, multiple acupuncture points are widely used, especially GB20 and Yanglingquan (GB34). However, the role played by vasoactive neurotransmitters in acupuncture antihyperalgesic effect at the single or multiple acupuncture points remains unknown. We aimed to determine whether EA would exert its antihyperalgesic effects by modulating vasoactive neurotransmitter release from the perivascular nerves. Furthermore, we examined whether targeting multiple acupuncture points would be more effective than targeting a single point in reducing hyperalgesia. The mechanical and thermal hyperalgesia were evaluated by measuring the facial and hind-paw mechanical withdrawal thresholds, tail-flick and hot-plate latencies. Plasma concentrations of vasoactive neurotransmitters were determined using rat-specific ELISA kits from jugular vein, including calcitonin gene-related peptide (CGRP), substance P (SP), vasoactive intestinal peptide (VIP), neuropeptide Y (NPY), pituitary adenylate cyclase-activating polypeptide (PACAP), nitric oxide (NO), and endothelin-1 (ET-1). The result suggested that EA significantly ameliorated the mechanical and thermal hyperalgesia, reduced c-Fos levels in the trigeminal ganglion, and attenuated plasma and dural levels of vasoactive neurotransmitters, especially in the multiple acupuncture points group (GB20+GB34). In conclusion, EA exerts antihyperalgesic effect in a rat model of conscious recurrent migraine, possibly via modulation of the vasoactive neurotransmitters. Furthermore, targeting multiple acupuncture points is more effective than targeting a single point in reducing hyperalgesia.

## 1. Introduction

Migraine is a perplexing, essentially inherited, and variable disorder of brain function that affects one in nine adults worldwide [1, 2]. It significantly affects an individual's quality of life [3], especially during peak years of productivity. Hyperalgesia or cutaneous allodynia, which refers to pain provoked by nonnociceptive stimulation, affects 39.9% of migraineurs [4]. The dysfunctional neurovascular interactions play a pivot role in migraine onset phase.

**Figure 1:**
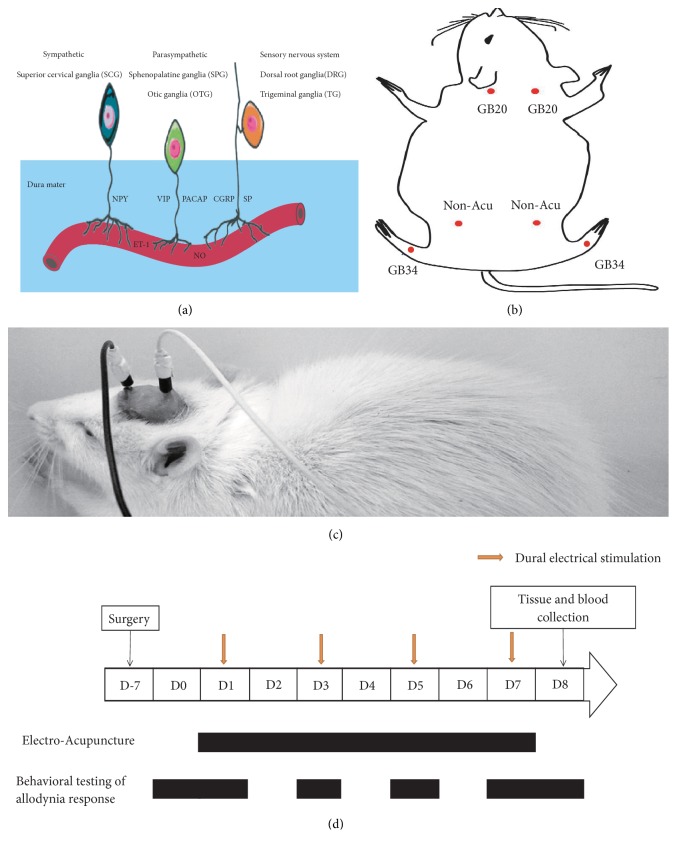
Overview of the dura mater and its associated constituents and diagram of the experimental protocol. (a) Overview of the dural vessels, perivascular nerves, and neurotransmitters. CGRP: calcitonin gene-related peptide; PACAP: pituitary adenylate cyclase-activating polypeptide; NO: nitric oxide; SP: substance P; NPY: neuropeptide Y; VIP: vasoactive intestinal peptide; ET-1: endothelin-1. (b) Rat schematic showing the location of the acupuncture points used in the present study. GB20: Fengchi, GB34: Yanglingquan; Non-Acu: nonacupuncture point. (c) Photograph of a conscious rat with a wire connection through the rubber tubing between the electrode and an electrical stimulator. (d) Rats received dural electrical stimulation (DES) every other day from Day 1 to Day 7 (on Days 1, 3, 5, and 7). Electroacupuncture (or nonacupuncture point acupuncture) was performed daily from Day 1 to Day 7 in each group following the DES. On the 7th day (Day 0) after surgery and the next day following the final DES session (Day 8), baseline and postintervention withdrawal thresholds were measured in conscious rats. On Days 1, 3, 5, and 7, facial and hind-paw withdrawal thresholds, tail-flick, and hot-plate latencies were measured in conscious rats following EA treatment. Tissue samples were collected after the final behavioral test.

Previous studies have suggested that vasodilation and contraction are associated with the development of migraine [5–8]. During the attack, a previous study has found that the contraction of extracerebral arteries is associated with the amelioration of headache [9]; and during the headache-free periods, it was found that the intracranial cerebral blood flow increases (vasodilatation) [10]. Moreover, during the migraine attack and the headache-free periods, the vasodilating neurotransmitters are elevated in the plasma, including calcitonin gene-related peptides (CGRP) [11], substance P (SP) [12, 13], vasoactive intestinal peptide (VIP) [14, 15], and nitric oxide (NO) [16]. At the same time, the vasoconstrictive neurotransmitters counteract a possible migraine-induced dilation. During the migraine attack, plasma neuropeptide Y (NPY) [17] and endothelin-1 (ET-1) level [18] are significantly increased relative to the levels in healthy individuals, while during the headache-free periods, the ET-1 level is significantly increased relative to the level in healthy individuals [18] and the plasma NPY level [19] is not significantly altered. These vasoconstrictive neurotransmitters are also important for regulating the peripheral and central circulation [20], leading to activation and sensitization of the trigeminovascular system (TS) (Figure 1(a)) [21–23].

A recent study has also shown that intracerebral vasodilatation is positively correlated with headache frequency and the number of cutaneous allodynia symptoms [10]. Cutaneous allodynia or hyperalgesia, which is defined as the perception of pain or discomfort in response to innocuous thermal (heat or cold) and/or mechanical (static or dynamic) stimuli, has been recognized as a clinical manifestation of central sensitization in migraine patients [24]. Furthermore, a preclinical experiment has suggested that the levels of vasoactive neurotransmitters CGRP and pituitary adenylate cyclase-activating polypeptide (PACAP) are dynamically elevated in the trigeminal ganglion (TG), and facial mechanical withdrawal threshold (MWT) gradually decreases as the number of dural electrical stimulations (DES) increases [5]. Therefore, these vasoactive neurotransmitters are key mediators in the pathophysiology of migraine [25, 26].

Acupuncture is known to be effective for alleviating pain and cutaneous allodynia and for preventing recurrent migraine [27, 28]. In our previous studies, we demonstrated that acupuncture could effectively alleviate mechanical hyperalgesia and normalizes the levels of abnormal biomarkers (c-Fos, CGRP, and serotonin [5-HT]) [29–31]. However, the peripheral trigeminovascular system has received little attention, especially with respect to vasoactive neurotransmitters and specific dural perivascular nerves. In addition, most previously preclinical studies utilized a single acupuncture point (e.g., GB20 [Fengchi]) for the treatment of migraine [29–31]. However, multiple acupuncture points are often used in clinical practice (e.g., GB20 and Yanglingquan [GB34]) [28, 32–35]. Therefore, the present study had two main aims. First, using a rat model of conscious recurrent migraine, we sought to determine whether electroacupuncture (EA) would exert an antihyperalgesic effect by modulating vasoactive neurotransmitter release from the dural perivascular nerves. Second, we examined whether acupuncture at multiple points would be more effective than that of a single point in attenuating hyperalgesia.

## 2. Methods

### 2.1. Animals

The present study was approved by the Institutional Review Board for Animal Experiments at Capital Medical University (approval number: AEEI 2015-075). Surgeries were performed under general anesthesia, and all possible efforts were made to minimize suffering. 60 eight-week-old male, specific-pathogen-free Sprague Dawley rats (Academy of Military Medical Sciences, SCXK-PLA 201200004, Chinese People's Liberation Army, China), weighing 210 ± 10 g, were used in this study. Rats were housed in groups of four in cages (64 cm × 40 cm × 22 cm) for 1 week prior to undergoing brain surgery, and then they were housed individually following electrode implantation. All animals were housed in an ambient laboratory environment (room temperature: 22°C; humidity: 40%-60%) and had free access to food and water, and were housed under a 12-h light/dark cycle.

### 2.2. Surgical Procedures

As described in a previous study [36], rats were anesthetized via an intraperitoneal injection of 60 mg/kg pentobarbital sodium (Sigma-Aldrich, St. Louis, MO, USA). Two holes (approximately 1 mm in diameter) were drilled using a dental drill (78001; RWD Life Science, Shenzhen, Guangdong Province, China). The drill was positioned 4 mm anterior and 6 mm posterior to the bregma and the midline, respectively. The cranial holes were located over the dura mater around the superior sagittal sinus (SSS). All rats were allowed a 7-day recovery period prior to the DES and EA treatment.

### 2.3. Animal Groups

Based on methods used previously [29], we included 10 animals in each group. After surgery, 60 animals were randomly divided into the following six groups: a sham-operated (Sham) group, which underwent electrode implantation only; a model (Model) group, which received DES only; a nonacupuncture point (Non-Acu) group, which included DES rats that received EA at a distant nonacupuncture point [37] (approximately 10 mm above the iliac crest); and GB20+GB34, GB20, and GB34 groups after DES, respectively. According to the World Health Organization Standard Acupuncture Point Locations (World Health Organization Regional Office for the Western Pacific, 2008), GB20 is located “in the anterior region of the neck, inferior to the occipital bone, in the depression between the origins of the sternocleidomastoid and the trapezius muscles.” The anatomical position of GB20 in the rat is located 3 mm from the midpoint of the two ear connecting lines behind the head [29]. The rat GB34, which is determined according to the GB34 position in humans, is located at the depression anterior and inferior to the head of the fibula [38, 39] (Figure 1(b)).

### 2.4. Induction of Recurrent Conscious Migraine in Rats via DES

The experiment started after recovery period and lasted for 8 days. DES was administered to the Non-Acu, GB20+GB34, GB20, GB34 and Model groups using a stimulator (YC-2 stimulator; Chengdu Instrument Factory, Chengdu, Sichuan Province, China) in conscious rats. In accordance with methods described previously, DES, which consisted of 0.5 ms monophasic square wave pulses of 1.8–2.0 mA (intensity) and 20 Hz (frequency), was administered to rats for a 15 min period every other day (on Days 1, 3, 5, and 7), for a total of four sessions [31] (Figure 1(c)).

### 2.5. EA Treatment

EA was applied at a frequency of 2/15 Hz (amplitude-modulated wave) and an intensity of 0.5–1.0 mA (depending on the reaction of the rat) for 15 min without anesthesia. From Day 1 to Day 7, the Non-Acu, GB20+GB34, GB20, and GB34 groups received EA following electrical stimulation for a total of seven sessions. Stainless-steel acupuncture needles (25 mm × 0.25 mm; Suzhou Medical Appliance Factory, Suzhou, Jiangsu Province, China) were bilaterally inserted into GB20 and GB34 at a depth of 2–3 mm (Figure 1(b)). However, in the Sham and Model groups, rats were held for 15 min, but no EA was applied.

### 2.6. Behavioral Testing of the Hyperalgesia Response

According to previous studies, decreases in the MWT and thermal withdrawal threshold (TWT) indicate the presence of hyperalgesia [31, 40]. The MWT can be measured using a von Frey anesthesiometer [31, 40], while the TWT can be measured via the hot-plate [41] and the tail-flick test [42]. In this study, an electronic von Frey anesthesiometer (Model 2390; IITC Life Science, Woodland Hills, CA, USA) was used to measure the facial (midline of the forehead) and hind-paw (plantar aspect) withdrawal thresholds. The pain response to thermal stimuli was assessed using the tail-flick and hot-plate analgesia meter (Yiyan Technology Development Co., Ltd., Jinan, Shandong Province, China). Measurements were performed at least 24 h before the experiment (baseline on Day 0), 0.5 h after treatment sessions (on Days 1, 3, 5, and 7), and 24 h after the last treatment session (on Day 8) (Figure 1(d)). All tests were performed in awake and freely moving animals, without any sedation. The experiment was evaluated by an investigator blinded to the experimental groups. A different investigator conducted the experiments [43].

Rats were first acclimated to the experimental environment and innocuous mechanical stimulation for 3 days before electrode implantation. The von Frey anesthesiometer probe was applied vertically to the skin of the face or hind-paw until the rat made an escape movement. When the escape response occurred, the maximum force was recorded as the withdrawal threshold. In accordance with preliminary observations and previous studies, rats with forces of <8 g [43] and >60 g [44] for the facial MWT and <15 g [43] and >60 g [44] for the hind-paw MWT at baseline were excluded from further analyses.

#### 2.6.1. Facial Hyperalgesia

Rats were placed in a tailored plastic restraint tube (length, 25 cm; inner diameter, 8 cm) with a mesh inlay at the front, which enabled easy access to the periorbital region. After a 30-min habituation period, the von Frey anesthesiometer tip was applied to the periorbital region with steady vertical pressure until an escape movement occurred. A total of three trials were completed at 30-s intervals. The mean value from all trials was considered the facial MWT.

#### 2.6.2. Hind-Paw Hyperalgesia

Rats were placed separately under transparent plastic boxes on an elevated mesh platform for 30 min. The von Frey anesthesiometry probe was inserted through the mesh to prod the hind-paw until the paw was withdrawn from the tip or lifted off the mesh floor. The assay was performed three times at 30-second intervals. The mean value from all trials was considered the hind-paw MWT.

#### 2.6.3. Tail-Flick Latency

Rats were placed separately in a rat frame for 30 min. Briefly, radiant heat was applied approximately 4 cm from the tip of the animal's tail. The intensity of the thermal stimulus was adjusted to provide an average baseline tail-flick latency of 3–4 s. A cut-off time of 16 s was set to avoid injury. The control reaction time and the latency of the response were recorded two times, with an interval of 5 min between readings. The mean value of all trials was considered the tail-flick latency.

#### 2.6.4. Hot-Plate Latency

In the hot-plate test, we measured the latency to lick a hind-paw when the rat was placed on a 50°C plate [45–47]. Rats were placed separately on a plate enclosed with four plexiglass walls (31 cm × 22 cm × 30 cm), which prohibited the rat from escaping. Rats were then moved to an adjacent room for the hot-plate test. The rat was removed from the plate immediately upon licking a hind-paw or if no response occurred within 50 s. The control reaction time and the latency of the response were recorded twice, with an interval of 5 min between readings. The mean value of all trials was considered the hot-plate latency.

### 2.7. Immunofluorescence and Image Analysis

After the last behavioral assessment, animals were anesthetized with 1% pentobarbital sodium (60 mg/kg) intraperitoneally, and intracardially perfused with 0.1 M phosphate-buffered saline (PBS), followed by 400–500 mL of 4% paraformaldehyde (PFA) in PBS. After fixation, rats were decapitated, and the head was divided into two symmetrical sections along the sagittal line. Except for the dura mater, all soft tissue was removed from the inside and outside of the skull. The skull with the attached dura mater was immersed in 4% PFA and fixed at 4°C for an additional 4 h. The skull was subsequently rinsed and stored in cold PBS. Within the next few days, the dura mater was removed from the skull using a scalpel along the edge of the tentorium, SSS, cranial fossa, and TG. The TG was removed and fixed in 4% PFA /PBS at 4°C overnight, after which it was transferred to 30% sucrose for 72 hours for cryopreservation. Following this, the cranial dura mater was carefully removed and processed as a whole mount for staining with an indirect immunofluorescence technique using a goat anti-CGRP antibody (ab36001, 1:100, Abcam, Cambridge, UK) and a rabbit anti-VIP antibody (ab22736, 1:400, Abcam) for 2 days. Rabbit anti-goat or goat anti-rabbit immunoglobulin G (IgG; 1:200) labeled with fluorescein isothiocyanate (RAG001, 1:200, MultiSciences Biotech Co., Ltd, Hangzhou, China) or Alexa 488 (A0432, 1:200, Beyotime Biotech Inc., Shanghai, China) was used as the secondary antibody for 4 h at 22°C. The dura sectors were incubated with primary antibodies for 2 days with gentle agitation at 4°C, following which they were incubated with secondary antibodies for 4 h at 22°C. A mixture of 10% bovine serum albumin with 0.3% Triton X-100 was used for preincubation (2 h) and rinses (2 h) between incubation with primary and secondary antibodies. To control for the uneven distribution of TG neurons, we prepared 3 slides per animal (20 *μ*m-thick sections/slide) at random. Slides were incubated with rabbit anti-c-Fos antibody (Ab190289, 1:5000, Abcam) overnight at 4°C, and then treated with Cy3-labeled goat anti-rabbit IgG (H+L) secondary antibodies (A0516, 1:200, Beyotime Biotech Inc.) for 1 h at 22°C before being mounted, and cover-slipped in antifade mounting medium with DAPI (H-1500, Vector Laboratories, Inc., Burlingame, CA). The images were captured using a Leica semiautomatic light microscope (Leica DM5500B). The CGRP-positive and VIP-positive fluorescence images were measured in squares at 20× magnification, and c-Fos-positive cells were counted at 40× magnification. Cell and nerve fiber densities in the TG and dura mater were calculated by a blinded assessor using Image J software.

### 2.8. Western Blotting

Rats were anesthetized with 1% pentobarbital sodium (60 mg/kg) intraperitoneally, and the TG was rapidly removed, placed into 1.8-mL prechilled tubes, frozen in liquid nitrogen, and stored at -80°C. For western blot assays, the TG was collected and homogenized in radioimmunoprecipitation assay buffer (70-WB019; MultiSciences Biotech Co. Ltd.) using an ultrasonic cell crusher. The homogenate was centrifuged at 13000 × g for 20 min at 4°C, and 300 *μ*L of the supernatant was collected and stored at -20°C until analysis. The protein concentrations of the samples were determined via a bicinchoninic acid assay using the Micro BCA Protein Assay Kit (Applygen Technologies Inc., Beijing, China). Proteins were separated via sodium dodecyl sulfate-polyacrylamide gel electrophoresis (10% gradient gels), with 50 *μ*g of protein per well, following which they were transferred to polyvinylidene difluoride membranes. The polyvinylidene difluoride blots were blocked in 10% bovine serum albumin for 1 h at 22°C. Membranes were incubated with either a rabbit anti-c-Fos polyclonal antibody (sc-447,1:500, Santa Cruz Biotechnology Inc, USA) or a mouse glyceraldehyde 3-phosphate dehydrogenase (GAPDH) monoclonal antibody (C1312, 1:1000, Applygen Technologies Inc.) overnight at 4°C. Peroxidase-conjugated affinipure goat anti-rabbit IgG (H+L), goat anti-rabbit IgG (H+L) (C1309, 1:10,000, Applygen Technologies Inc.), or peroxidase-conjugated affinipure goat anti-mouse IgG (H+L) (C1308, 1:10,000, Applygen Technologies Inc.) was diluted in 10% bovine serum albumin/Tris-buffered saline/Tween 20 (TBST) prior to use at 22°C. All between-incubation washes were performed using TBST. Signals were detected using an enhanced chemiluminescence kit (Applygen Technologies Inc.) and Kodak film (Eastman Kodak, Rochester, NY, USA). The integrated optical density values of the detected proteins were analyzed using Image J software. The level of c-Fos was expressed as a ratio to GAPDH (loading control).

### 2.9. Sampling and Biochemical Analysis

At the end of all experiments, animals were deeply anesthetized with 60 mg/kg pentobarbital sodium, and blood samples were collected from the external jugular vein. The samples were then mixed with ethylenediaminetetraacetic acid, centrifuged at 4000 rpm for 10 min at 4°C, and stored at −20°C until further analysis. Plasma concentrations of SP, CGRP, PACAP, VIP, NPY, NO, and ET-1 were measured using rat-specific enzyme-linked immunosorbent assay (ELISA) kits (R&D, USA; Spibio, USA; Raybio, USA; Aviva Systems Biology, USA), in accordance with the manufacturer instructions.

### 2.10. Statistical Analysis

The results were presented as the means ± standard deviations (SD). All analyses were performed using SPSS version 18.0. Significant differences in the MWT and TWT were assessed using two-way (time, intervention) repeated-measures analyses of variance (ANOVA). When the interaction between intervention and time resulted in a* P* value less than 0.05, the data were analyzed using a one-way ANOVA. Multiple comparisons were conducted using the Bonferroni test (equal variances assumed) or Tamhane test (equal variances not assumed). In all cases, the level of statistical significance was set at* P*<0.05.

## 3. Results

### 3.1. Electroacupuncture Attenuated Hyperalgesia in Rats with Recurrent Conscious Migraine

In the present study, we investigated whether EA would alleviate the gradual reductions in MWT and TWT following DES, and whether EA at multiple acupuncture points would be more effective at attenuating hyperalgesia than EA at a single point. To achieve our aims, we measured MWTs in the face and hind-paw, as well as hot-plate and tail-flick latency at least 30 min after EA.

**Figure 2:**
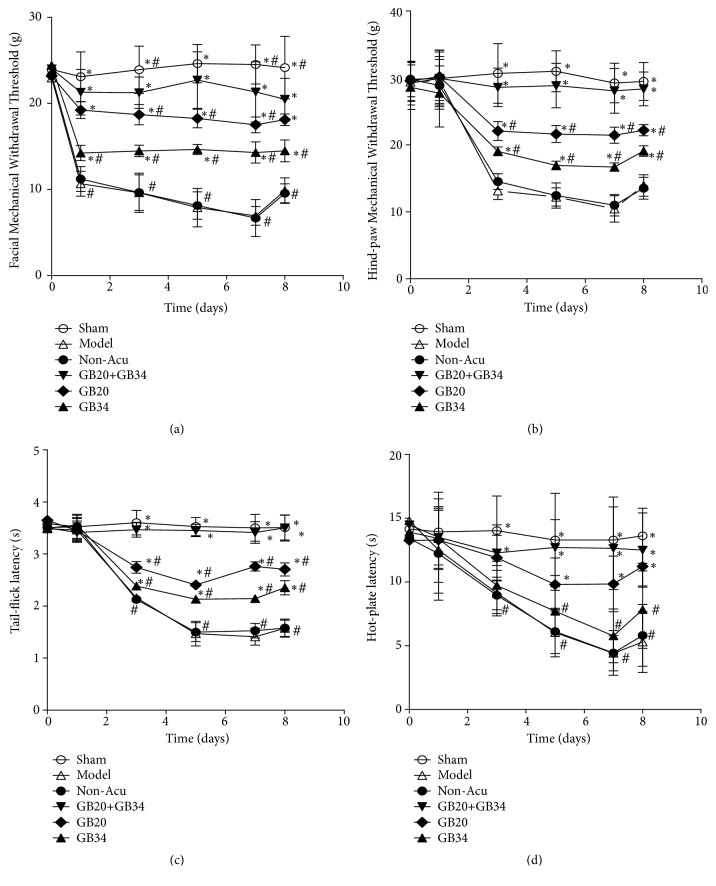
Electroacupuncture (EA) treatment alleviated impairments in the mechanical withdrawal thresholds of the face (a) and hind-paw (b) and prolonged tail-flick (c) and hot-plate (d) latencies in rats subjected to dural electrical stimulation. Data are presented as the mean ± standard deviation, n=10/group; ∗Sham, GB20+GB34, GB20, and GB34 group vs. Model group (∗*P*<0.05); # Sham, Non-Acu, GB20, and GB34 group vs. GB20+GB34 group (#*P*<0.05).

For the facial and hind-paw MWTs, we observed significant effects of time (F [4.098]=130.885,* P*<0.001; F [3.641]=164.292,* P*<0.001), and intervention (F [5]=388.387,* P*<0.001; F [5]=321.016,* P*<0.001), and significant interaction effects between intervention and time (F [20.488]=15.366,* P*<0.001; F [18.205]=19.420,* P*<0.001). On Days 3, 5, 7, and 8, the facial and hind-paw MWTs were lower in Model group than in the Sham group (*P*<0.05). Following EA treatment, the DES-induced reductions in facial MWTs (GB20+GB34, P<0.05; GB20,* P*<0.05; GB34,* P*<0.05) were significantly increased on Days 1, 3, 5, 7 and 8, while those in hind-paw MWTs (GB20 + GB34,*P*<0.05; GB20,* P*<0.05; GB34,* P*<0.05) were significantly increased on Days 3, 5, 7, and 8. Notably, the elevations in MWTs were greater in the GB20+GB34 group than in the GB20 (*P*<0.05) and GB34 (*P*<0.05) groups on Days 3, 5, 7, and 8. No significant differences in facial or hind-paw MWTs were observed between the Non-Acu and Model groups at any time (all* P*>0.05; Figures 2(a) and 2(b)).

For tail-flick and hot-plate latencies, we observed significant effects of time (F [4.215]=525.886,* P*<0.001; F [5] = 54.972,* P*<0.001), and intervention (F [5]=534.624, P<0.001; F [5]=35.031,* P*<0.001), and significant interaction effects between intervention and time (F [21.075]=65.185,* P*<0.001; F [25]=5.105,* P*<0.001). On Days 3, 5, 7, and 8, tail-flick and hot-plate latencies were greater in Model group than in Sham group. Following EA treatment, the DES-induced reductions in tail-flick (GB20+GB34,* P*<0.001; GB20,* P*<0.05; GB34,* P*<0.05) and hot-plate (GB20+GB34,* P*<0.05; GB20,* P*<0.05 (on Days 5, 7 and 8); GB34,* P*>0.05) latencies were significantly attenuated. Furthermore, the latencies of the GB20+GB34 group is significantly longer than those of the GB20 (*P*<0.05) and GB34 (*P*<0.05) groups on Days 3, 5, 7, and 8 in the tail-flick test, and than those of the GB34 group (*P*<0.05) on Days 5, 7, and 8 in the hot-plate test. No significant differences in tail-flick or hot-plate latencies were observed between the Non-Acu and Model groups at any time point (all* P*>0.05; Figures 2(c) and 2(d)).

Overall, these findings indicated that EA attenuated hyperalgesia in rats following DES, and that the therapeutic effects of EA stimulation at GB20+GB34 are superior those observed for EA at a single point (GB20 or GB34).

### 3.2. EA Reduced c-Fos Levels in the TG in a Rat Model of Conscious Migraine

**Figure 3:**
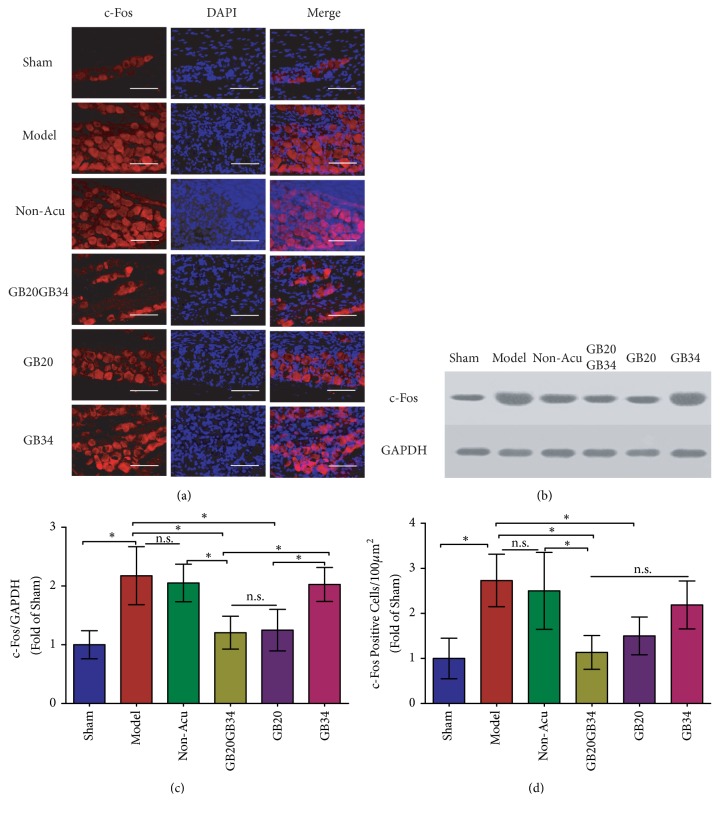
The number of c-Fos positive cells (a, d) and levels of c-Fos protein (b, c) were compared in the trigeminal ganglion among the six groups. Data are presented as the mean ± standard deviation, n=5/group. Scale bar = 50 *μ*m. ∗*P*<0.05.

DES induced elevations in c-Fos expression in the TG. C-Fos expression is regarded as a biomarker of pain-related behavior, reflecting the degree of hyperalgesia and the level of activation of the trigeminovascular system (TS) [29, 48–50]. To investigate the effects of EA on hyperalgesia, we performed immunofluorescence analyses and western blotting to examine the number of c-Fos-positive cells and levels of c-Fos protein in the TG (Figure 3). The number of c-Fos-positive cells (Figures 3(a) and 3(d)) and levels of c-Fos protein (Figures 3(b) and 3(c)) in the TG were significantly more and higher in the Model group (after repeated DES) than in the Sham group (n=5,* P*<0.05 for both). In contrast, the EA-treated groups (except GB34) exhibited significantly fewer c-Fos-positive cells (n=5,* P*<0.05) and significantly lower c-Fos protein levels than the Model group (n=5,* P*<0.05), especially the GB20+GB34 group. The c-Fos positive cell numbers and c-Fos protein levels in the Non-Acu group did not significantly differ from those in the Model group (*P*>0.05 for both).

### 3.3. EA Reduced the Plasma Concentrations of Vasoactive Neurotransmitters

#### 3.3.1. EA Reduced Plasma Concentrations of CGRP and SP

**Figure 4:**
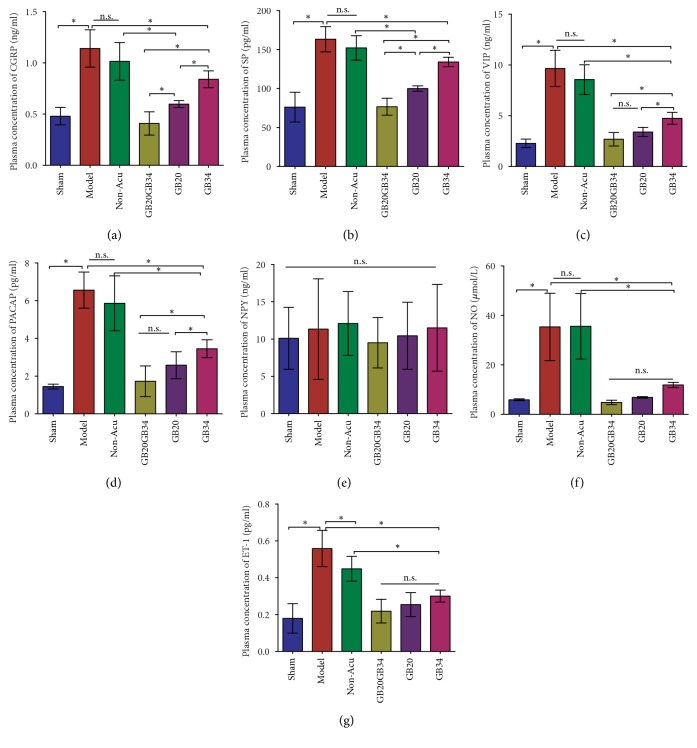
Plasma levels of vasoactive neurotransmitters in the six groups. Vasoactive neurotransmitter release was significantly higher in Model group rats with migraine than in those of the sham-operated group. Electroacupuncture reduced the release of CGRP (a), SP (b), VIP (c), PACAP (d), NO (f), and ET-1 (g), while nonacupuncture point treatment had no effect on the levels of these vasoactive neurotransmitters in rats with migraine. No variations in plasma NPY levels were observed among the six groups. Data are presented as the mean ± standard deviation, n=10/group. ∗*P*<0.05. CGRP: calcitonin gene-related peptide; PACAP: pituitary adenylate cyclase-activating polypeptide; NO: nitric oxide; SP: substance P; NPY: neuropeptide Y; VIP: vasoactive intestinal peptide; ET-1: endothelin-1.

The ELISA results revealed that DES (Model group) significantly elevated the plasma levels of CGRP (Figure 4(a)) and SP (Figure 4(b)) relative to those observed in the Sham group (*P*<0.001 for both). When compared with the levels observed in the Model group, the plasma levels of CGRP and SP in the EA-treated (GB20+GB34, GB20, GB34) groups (*P*<0.001 for all) were significantly reduced; however, no significant differences in CGRP and SP levels were observed between the Model and Non-Acu group (*P*>0.05 for both). Furthermore, the GB20+GB34 group exhibited significantly lower plasma levels of CGRP and SP levels than did the GB20 (*P*<0.05,* P*<0.01) or GB34 (*P*<0.001,* P*<0.001) group. In addition, plasma CGRP and SP levels were significantly lower in the GB20+GB34 (*P*<0.001) and GB20 (*P*<0.001) groups than in the Non-Acu group.

#### 3.3.2. EA Reduced Plasma Concentrations of VIP and PACAP

To evaluate the effects of EA at GB20+GB34, GB20, and GB34 on parasympathetic nerve activation in our rat model, we assessed plasma levels of VIP and PACAP release from parasympathetic nerves. Our ELISA results indicated that the Model group had significantly elevated plasma levels of VIP (Figure 4(c)) and PACAP (Figure 4(d)), relative to those observed in the Sham group. However, the EA-treated (GB20+GB34, GB20, GB34) groups exhibited significantly reduced plasma VIP and PACAP levels, relative to those observed in the Model group. No significant difference in the reduction of plasma VIP concentration were observed between the GB20 (*P*<0.01) and GB20+GB34 (*P*<0.01) groups. And, the reductions in VIP and PACAP levels were greater in the GB20+GB34 group than in the GB34 group (*P*<0.01). No significant difference in the VIP or PACAP level was identified between the Model and Non-Acu groups. Furthermore, plasma VIP levels were lower in the GB20+GB34 (*P*<0.01), GB20 (*P*<0.01), and GB34 (*P*<0.01) groups than in the Non-Acu group.

#### 3.3.3. EA Did Not Affect Plasma Concentrations of NPY

Repeated DES did not significantly alter plasma levels of NPY released from sympathetic nerves (Figure 4(e)). Furthermore, no significant differences in plasma NPY levels were observed among the Non-Acu, GB20+GB34, GB20, and GB34 groups.

#### 3.3.4. EA Attenuated the Plasma Levels of NO and ET-1

Plasma concentrations of NO (Figure 4(f)) and ET-1 (Figure 4(g)) were significantly higher in the Model group than in the Sham group (*P*<0.001). However, the NO and ET-1 levels of EA-treated groups were significantly lower than those in the Model group (*P*<0.001 for all) with no significant differences in the levels being identified among the GB20+GB34, GB20, and GB34 groups (*P*>0.05). Plasma levels of NO and ET-1 did not significantly differ between the Non-Acu and Model groups (*P*>0.05 for both).

### 3.4. Acupuncture Attenuated the DES-Induced Reductions in Dural CGRP and VIP Expression

**Figure 5:**
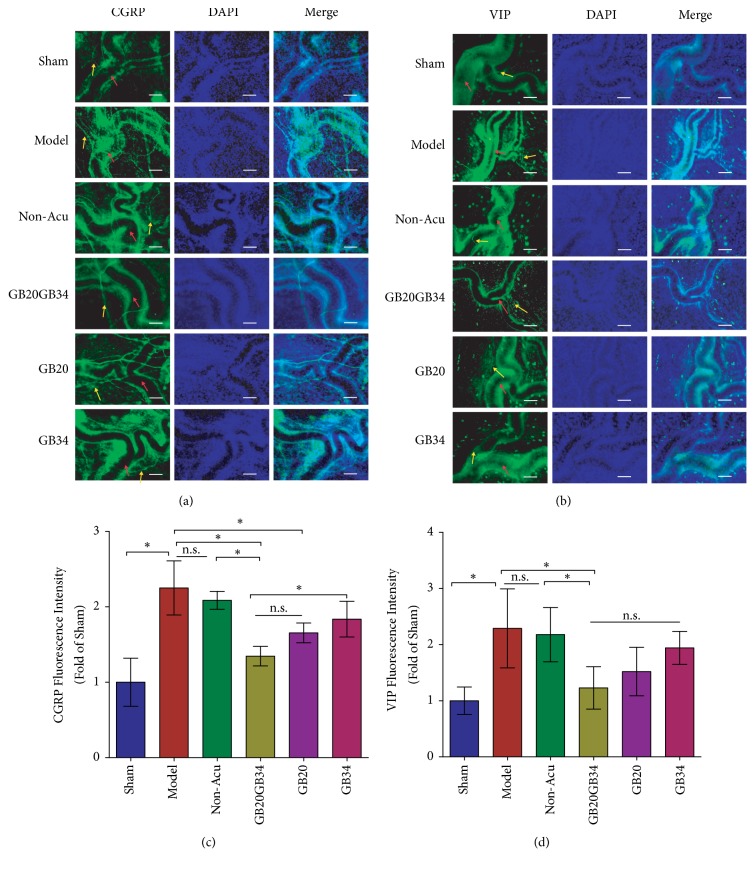
Dural fluorescence intensity of the vasoactive neurotransmitters (CGRP and VIP) in the six groups. Fluorescence intensity for vasoactive neurotransmitters was significantly higher in Model group rats with migraine than in those of the sham-operated group rats. Electroacupuncture reduced the intensity of CGRP (yellow arrowheads in (a)) (a, c) and VIP (yellow arrowheads in (b)) (b, d) expression, while nonacupuncture point treatment had no effect on the levels of these vasoactive neurotransmitters in rats with migraine. The red arrowheads in (a, b) represent the middle meningeal artery. Data are presented as the mean ± standard deviation, n=5/group. Scale bar = 50 *μ*m. ∗*P*<0.05. CGRP: calcitonin gene-related peptide; VIP: vasoactive intestinal peptide.

To determine whether EA influences the expression of vasoactive neurotransmitters in the dura mater, we performed immunofluorescence analyses of CGRP and VIP expression. The expression levels of CGRP and VIP were significantly elevated in Model than that of Sham group (*P*<0.001 for both; Figure 5). The CGRP level in the GB20+GB34 and GB20 groups, but not the GB34 group, was significantly reduced relative to that in the Model group, while only the GB20+GB34 group showed significant reductions in the VIP level relative to that in the Model group (P<0.05; Figure 5). No significant differences in CGRP or VIP expression were observed between the Model and Non-Acu groups (*P*>0.05; Figures 5(b) and 5(d)), or between the GB20+GB34 and GB20 groups (*P*>0.05; Figures 5(b) and 5(d)).

## 4. Discussion

To the best of our knowledge, the present study is the first to investigate the effects of EA at a single acupuncture point (GB20 and GB34 alone) or multiple acupuncture points (GB20+GB34) on hyperalgesia and vasoactive neurotransmitter release from the perivascular nerves, which includes the sympathetic, parasympathetic, and sensory nerves, in a rat migraine model. We found that EA stimulation at GB20+GB34, GB20, or GB34 reversed mechanical and thermal sensitization, attenuated DES-induced activation of the trigeminovascular system (c-Fos), and decreased plasma and dural levels of vasoactive neurotransmitters. Furthermore, it was found that the antihyperalgesia effect of EA at GB20+GB34 group was better than that of GB20 or GB34 group. These findings suggested that EA attenuated hyperalgesia by modulating the release of vasoactive neurotransmitters, and that EA at multiple acupuncture points was more effective than EA at a single point in a rat model of recurrent migraine.

Our results suggested that EA could improve peripheral and central sensitization following recurrent DES. Central and peripheral sensitization is a crucial process underlying the increases in central and peripheral neuronal excitability [51]. Central sensitization manifests as hyperalgesia, particularly dynamic tactile allodynia [52]. Cephalic allodynia is the presence of sensitization of second-order neurons in the spinal trigeminal nucleus [53], which can be measured with the facial MWT [31, 40]. Moreover, whole-body allodynia is the presence of sensitization of the thalamus [54], which can be measured with the hind-paw MWT [31, 40] and hot-plate [41] and tail-flick latencies [42]. In accordance with our findings, several previous studies have demonstrated that repetitive DES results in gradual worsening and spreading of hyperalgesia [5]. In our study, elevations in the level of c-Fos protein expression and the number of c-Fos-positive cells were observed in the TG. Such elevations may be associated with peripheral sensitization, while reductions in the MWT and TWT induced by DES may reflect central sensitization. In addition, the previous researches have indicated that EA can elicit antihyperalgesic (elevated MWTs) effects in rats with DES-induced migraine [31, 40]. Moreover, in the present study, EA not only increased MWTs, but also improved TWTs.

Previous researches had suggested that the release of vasoconstrictive neurotransmitters led to activation and sensitization of the trigeminovascular system [21–23]. Vasoactive neurotransmitters play a causative role in migraine, reflecting a secondary event arising from the activation of trigeminal afferents that ensures the meningeal release of additional vasoactive neurotransmitters [55, 56]. Several studies have demonstrated that vasodilation neurotransmitters are released upon stimulation of the trigeminal nerve, causing vasodilation of dural vessels, thereby leading to perivascular changes, activation/sensitization of the trigeminovascular system, and the release of vasoactive neurotransmitters CGRP [12, 57], SP [12, 57], VIP [58], PACAP [59] from the parasympathetic nerves (sphenopala tine ganglia and otic ganglia) and sensory nerves (TG, and dorsal root ganglion) [60]. However, the plasma NPY [58] levels are not changed from sympathetic nerves (superior cervical ganglia) following stimulation of the trigeminal nerve. (Figure 1(c)). During activation of the trigeminovascular system, CGRP and SP induce vasodilatation and plasma protein extravasation in the meningeal vasculature, leading to mast cell degranulation followed by peripheral sensitization [61–64]. In addition, PACAP and NO play key roles in sensitization [65, 66] and neuroinflammation [67]. Consistent with previous findings, we observed increases in plasma levels of vasoactive neurotransmitters (except NPY) in migraine rats.

The result of the present study suggested that EA can restore the plasma and dural levels of vasoactive neurotransmitters to normal following DES. We evaluated the plasma concentrations of CGRP, SP, VIP, PACAP, NPY, NO, and ET-1 and the dural levels of CGRP and VIP to explore how EA regulates vasoactive neurotransmitters. Our experiments revealed that EA significantly reduced the DES-induced overexpression of CGRP, SP, VIP, PACAP, NO and ET-1 in recurrent migraine model rats. Similarly, in some studies, EA reduced the level of SP [68], CGRP [69], VIP [70–72], NPY [73], NO [74] and ET-1 [75] in plasma or some tissues of other diseases model animals, including dorsal root ganglion, liver, synovial, distal colon, spinal cord, hypothalamus, colonic mucosa, and cardiac tissue. However, we did not observe any effects of EA on the plasma concentrations of NPY. A possible reason is that the plasma NPY level did not change following DES. Therefore, EA had a comprehensive regulating effect on vasoactive neurotransmitters secretion in different models.

Combinations of multiple acupuncture points are generally used for the acupuncture treatment of migraine in clinical practice, especially GB20 and GB34 [28, 32, 34, 35, 76]. Moreover, EA at Waiguan (SJ5) and GB34 decreases glutamate levels and increases lipid levels in migraine model rats [77]. Indeed, the vast majority of preclinical studies focused on the single acupuncture point (GB20). EA at GB20 has been shown to ameliorate the changes in the MWT by altering c-Fos, 5-HT, 5-HT7R, CGRP, and cannabinoid 1 receptor (CB1R) expression in the descending pathway of migraine models [29–31, 40, 78]. However, few studies have investigated the molecular mechanisms underlying EA at multiple acupuncture points, especially GB20 and GB34. In our study, it was found that the EA at multiple acupuncture points was more effective than that at a single point in attenuating hyperalgesia.

The study had limitations that should be pointed out. Previous preclinical and clinical studies reported that some brain regions exhibited atypical functional connectivity in migraineurs and migraine model rats, including somatosensory pain processing [79] (somatosensory cortex and posterior insula), emotional processing [80] (anterior insula, anterior cingulate cortex, and amygdala), cognitive components and memories of pain processing [81] (hippocampus and parahippocampal gyrus), and pain modulation [82] (periaqueductal grey). Moreover, these changes were associated with the development of hyperalgesia in migraine models [80]. However, our study only focused on pain modulation. In future studies, we will evaluate the effects of EA on the memory and emotional processing of pain. In addition, previous research has indicated that levels of vasoactive neurotransmitters change dynamically during the development of migraine-related hyperalgesia [5]. In our study, we only examined the levels of vasoactive neurotransmitters at the end of EA treatment. Thus, further studies are required to elucidate the dynamic changes in vasoactive neurotransmitters that occur during the entire experiment period.

## 5. Conclusion

Our findings demonstrated that the antihyperalgesic effect of EA on migraine may be mediated via the modulation of vasoactive neurotransmitters. In addition, our results support that EA at multiple acupuncture points may be more effective than EA at a single point for relieving hyperalgesia.

## Data Availability

The data used to support the findings of this study are available from the corresponding author upon request.

## References

[1] Lipton R. B., Bigal M. E., Diamond M., Freitag F., Reed M. L., Stewart W. F. (2007). Migraine prevalence, disease burden, and the need for preventive therapy. *Neurology*.

[2] Lipton R. B., Stewart W. F., Diamond S., Diamond M. L., Reed M. (2001). Prevalence and burden of migraine in the United states: data from the american migraine study II. *Headache*.

[3] Buse D. C., Manack A., Serrano D., Turkel C., Lipton R. B. (2010). Sociodemographic and comorbidity profiles of chronic migraine and episodic migraine sufferers. *Journal of Neurology, Neurosurgery & Psychiatry*.

[4] Dodick D. W., Reed M. L., Fanning K. M. (2019). Predictors of allodynia in persons with migraine: Results from the Migraine in America symptoms and treatment (MAST) study. *Cephalalgia*.

[5] Zhang Q., Han X., Wu H. (2019). Dynamic changes in CGRP, PACAP, and PACAP receptors in the trigeminovascular system of a novel repetitive electrical stimulation rat model: relevant to migraine. *Molecular Pain*.

[6] Hestehave S., Munro G., Pedersen T. B., Abelson K. S. P. (2017). Antinociceptive effects of voluntarily ingested buprenorphine in the hot-plate test in laboratory rats. *Laboratory Animals*.

[7] Hodkinson D. J., Veggeberg R., Kucyi A. (2016). Cortico–cortical connections of primary sensory areas and associated symptoms in migraine. *eNeuro*.

[8] Wang X., Fang Y., Liang J., Yan M., Hu R., Pan X. (2014). 5-HT7 receptors are involved in neurogenic dural vasodilatation in an experimental model of migraine. *Journal of Molecular Neuroscience*.

[9] Asghar M. S., Hansen A. E., Amin F. M. (2011). Evidence for a vascular factor in migraine. *Annals of Neurology*.

[10] Hodkinson D. J., Veggeberg R., Wilcox S. L. (2015). Primary somatosensory cortices contain altered patterns of regional cerebral blood flow in the interictal phase of migraine. *PLoS ONE*.

[11] Fan P., Kuo P., Lee M. T., Chang S., Chiou L. (2019). Plasma calcitonin gene-related peptide: a potential biomarker for diagnosis and therapeutic responses in pediatric migraine. *Frontiers in Neurology*.

[12] Fusayasu E., Kowa H., Takeshima T., Nakaso K., Nakashima K. (2007). Increased plasma substance P and CGRP levels, and high ACE activity in migraineurs during headache-free periods. *PAIN*.

[13] Jang M.-U., Park J.-W., Kho H.-S., Chung S.-C., Chung J.-W. (2011). Plasma and saliva levels of nerve growth factor and neuropeptides in chronic migraine patients. *Oral Diseases*.

[14] Cernuda-Morollón E., Martínez-Camblor P., Alvarez R., Larrosa D., Ramón C., Pascual J. (2015). Increased VIP levels in peripheral blood outside migraine attacks as a potential biomarker of cranial parasympathetic activation in chronic migraine. *Cephalalgia*.

[15] Goadsby P. J., Edvinsson L., Ekman R. (1990). Vasoactive peptide release in the extracerebral circulation of humans during migraine headache. *Annals of Neurology*.

[16] Uzar E., Evliyaoglu O., Toprak G. (2011). Increased asymmetric dimethylarginine and nitric oxide levels in patients with migraine. *The Journal of Headache and Pain*.

[17] Gallai V., Sarchielli P., Trequattrini A., Paciaroni M., Usai F., Palumbo R. (1994). Neuropeptide Y in juvenile migraine and tension‐type headache. *Headache*.

[18] Gallai V., Sarchielli P., Firenze C. (1994). Endothelin 1 in migraine and tension‐type headache. *Acta Neurologica Scandinavica*.

[19] Vécsei L., Widerlöv E., Ekman R. (1992). Suboccipital cerebrospinal fluid and plasma concentrations of somatostatin, neuropeptide Y and beta-endorphin in patients with common migraine. *Neuropeptides*.

[20] Faraci F. M. (1989). Effects of endothelin and vasopressin on cerebral blood vessels. *American Journal of Physiology-Heart and Circulatory Physiology*.

[21] Petersen K. A., Birk S., Doods H., Edvinsson L., Olesen J. (2004). Inhibitory effect of BIBN4096BS on cephalic vasodilatation induced by CGRP or transcranial electrical stimulation in the rat. *British Journal of Pharmacology*.

[22] Williamson D. J., Hargreaves R. J., Hill R. G., Shepheard S. L. (1997). Sumatriptan inhibits neurogenic vasodilation of dural blood vessels in the anaesthetized rat—Intravital microscope studies. *Cephalalgia*.

[23] Ebersberger A., Averbeck B., Messlinger K., Reeh P. W. (1999). Release of substance P, calcitonin gene-related peptide and prostaglandin E2 from rat dura mater encephali following electrical and chemical stimulation in vitro. *Neuroscience*.

[24] Tietjen G. E., Brandes J. L., Peterlin B. L. (2009). Allodynia in migraine: Association with comorbid pain conditions. *Headache: The Journal of Head and Face Pain*.

[25] Bellamy J. L., Cady R. K., Durham P. L. (2006). Salivary levels of CGRP and VIP in rhinosinusitis and migraine patients. *Headache: The Journal of Head and Face Pain*.

[26] Juhasz G., Zsombok T., Jakab B., Nemeth J., Szolcsanyi J., Bagdy G. (2005). Sumatriptan causes parallel decrease in plasma calcitonin gene-related peptide (CGRP) concentration and migraine headache during nitroglycerin induced migraine attack. *Cephalalgia*.

[27] Linde K., Allais G., Brinkhaus B. (2016). Acupuncture for the prevention of episodic migraine. *Cochrane Database of Systematic Reviews*.

[28] Wang L.-P., Zhang X.-Z., Guo J. (2011). Efficacy of acupuncture for migraine prophylaxis: a single-blinded, double-dummy, randomized controlled trial. *PAIN*.

[29] Pei P., Liu L., Zhao L., Cui Y., Qu Z., Wang L. (2016). Effect of electroacupuncture pretreatment at GB20 on behaviour and the descending pain modulatory system in a rat model of migraine. *Acupuncture in Medicine*.

[30] Liu L., Pei P., Zhao L.-P., Qu Z.-Y., Zhu Y.-P., Wang L.-P. (2016). Electroacupuncture pretreatment at GB20 exerts antinociceptive effects via peripheral and central serotonin mechanism in conscious migraine rats. *Evidence-Based Complementary and Alternative Medicine*.

[31] Zhao L.-P., Liu L., Pei P., Qu Z.-Y., Zhu Y.-P., Wang L.-P. (2017). Electroacupuncture at Fengchi (GB20) inhibits calcitonin gene-related peptide expression in the trigeminovascular system of a rat model of migraine. *Neural Regeneration Research*.

[32] Li Y., Liang F., Yang X. (2009). Acupuncture for treating acute attacks of migraine: a randomized controlled trial. *Headache*.

[33] Li Y., Zheng H., Witt C. M. (2012). Acupuncture for migraine prophylaxis: a randomized controlled trial. *Canadian Medical Association Journal*.

[34] Wang L.-P., Zhang X.-Z., Guo J. (2012). Efficacy of acupuncture for acute migraine attack: a multicenter single blinded, randomized controlled trial. *Pain Medicine*.

[35] Zhao L., Chen J., Li Y. (2017). The long-term effect of acupuncture for migraine prophylaxis: a randomized clinical trial. *JAMA Internal Medicine*.

[36] Dong Z., Jiang L., Wang X., Wang X., Yu S. (2011). Nociceptive behaviors were induced by electrical stimulation of the dura mater surrounding the superior sagittal sinus in conscious adult rats and reduced by morphine and rizatriptan benzoate. *Brain Research*.

[37] Li Q., Shi G., Yang J. (2015). Hippocampal cAMP/PKA/CREB is required for neuroprotective effect of acupuncture. *Physiology & Behavior*.

[38] Yim Y.-K., Lee H., Hong K.-E. (2006). Hepatoprotective effect of manual acupuncture at acupoint GB34 against CCl4-induced chronic liver damage in rats. *World Journal of Gastroenterology*.

[39] Siu F. K. W., Lo S. C. L., Leung M. C. P. (2005). Electro-acupuncture potentiates the disulphide-reducing activities of thioredoxin system by increasing thioredoxin expression in ischemia-reperfused rat brains. *Life Sciences*.

[40] Pei P., Liu L., Zhao L. P. (2019). Electroacupuncture exerts an anti-migraine effect via modulation of the 5-HT7 receptor in the conscious rat. *Acupuncture in Medicine*.

[41] Galeotti N., Ghelardini C. (2013). Inhibition of the PKC*γ*-*ε* pathway relieves from meningeal nociception in an animal model: an innovative perspective for migraine therapy?. *Neurotherapeutics*.

[42] Greco R., Mangione A. S., Sandrini G., Nappi G., Tassorelli C. (2014). Activation of CB2 receptors as a potential therapeutic target for migraine: evaluation in an animal model. *The Journal of Headache and Pain*.

[43] Boyer N., Dallel R., Artola A., Monconduit L. (2014). General trigeminospinal central sensitization and impaired descending pain inhibitory controls contribute to migraine progression. *PAIN*.

[44] Reali C., Fossat P., Landry M., Russo R. E., Nagy F. (2011). Intrinsic membrane properties of spinal dorsal horn neurones modulate nociceptive information processing in vivo. *The Journal of Physiology*.

[45] Pasban E., Panahpour H., Vahdati A. (2017). Early oxygen therapy does not protect the brain from vasogenic edema following acute ischemic stroke in adult male rats. *Scientific Reports*.

[46] Singh P., Kongara K., Harding D. (2018). Comparison of electroencephalographic changes in response to acute electrical and thermal stimuli with the tail flick and hot plate test in rats administered with opiorphin. *BMC Neurology*.

[47] Gunn A., Bobeck E. N., Weber C., Morgan M. M. (2011). The influence of non-nociceptive factors on hot-plate latency in rats. *The Journal of Pain*.

[48] Liu C., Zhang Y., Liu Q. (2018). P2X4-receptor participates in EAAT3 regulation via BDNF-TrkB signaling in a model of trigeminal allodynia. *Molecular Pain*.

[49] Long T., He W., Pan Q. (2018). Microglia P2X4 receptor contributes to central sensitization following recurrent nitroglycerin stimulation. *Journal of Neuroinflammation*.

[50] Su M., Ran Y., He Z. (2018). Inhibition of toll-like receptor 4 alleviates hyperalgesia induced by acute dural inflammation in experimental migraine. *Molecular Pain*.

[51] Goadsby P. J., Holland P. R., Martins-Oliveira M., Hoffmann J., Schankin C., Akerman S. (2017). Pathophysiology of migraine: a disorder of sensory processing. *Physiological Reviews*.

[52] Woolf C. J. (2011). Central sensitization: Implications for the diagnosis and treatment of pain. *PAIN*.

[53] Burstein R., Cutrer M. F., Yarnitsky D. (2000). The development of cutaneous allodynia during a migraine attack clinical evidence for the sequential recruitment of spinal and supraspinal nociceptive neurons in migraine. *Brain*.

[54] Burstein R., Jakubowski M., Garcia-Nicas E. (2010). Thalamic sensitization transforms localized pain into widespread allodynia. *Annals of Neurology*.

[55] Asghar M. S., Hansen A. E., Kapijimpanga T. (2010). Dilation by CGRP of middle meningeal artery and reversal by sumatriptan in normal volunteers. *Neurology*.

[56] Thomaides T., Karagounakis D., Spantideas A., Katelanis S. (2003). Transcranial Doppler in migraine attacks before and after treatment with oral zolmitriptan or sumatriptan. *Headache: The Journal of Head and Face Pain*.

[57] Mahmoudi J., Mohaddes G., Erfani M. (2018). Cerebrolysin attenuates hyperalgesia, photophobia, and neuroinflammation in a nitroglycerin-induced migraine model in rats. *Brain Research Bulletin*.

[58] Zagami A. S., Goadsby P. J., Edvinsson L. (1990). Stimulation of the superior sagittal sinus in the cat causes release of vasoactive peptides. *Neuropeptides*.

[59] Sándor K., Bölcskei K., McDougall J. J. (2009). Divergent peripheral effects of pituitary adenylate cyclase-activating polypeptide-38 on nociception in rats and mice. *PAIN*.

[60] Levy D., Labastida-Ramirez A., MaassenVanDenBrink A. (2018). Current understanding of meningeal and cerebral vascular function underlying migraine headache. *Cephalalgia*.

[61] Bigal M. E., Walter S., Rapoport A. M. (2013). Calcitonin gene-related peptide (CGRP) and migraine current understanding and state of development. *Headache*.

[62] Lennerz J. K., Rühle V., Ceppa E. P. (2010). Calcitonin receptor-like receptor (CLR), receptor activity-modifying protein 1 (RAMP1), and calcitonin gene-related peptide (CGRP) immunoreactivity in the rat trigeminovascular system: differences between peripheral and central CGRP receptor distribution. *Journal of Comparative Neurology*.

[63] Messlinger K., Fischer M. J. M., Lennerz J. K. (2011). Neuropeptide effects in the trigeminal system: Pathophysiology and clinical relevance in migraine. *The Keio Journal of Medicine*.

[64] Raddant A. C., Russo A. F. (2011). Calcitonin gene-related peptide in migraine: intersection of peripheral inflammation and central modulation. *Expert Reviews in Molecular Medicine*.

[65] Zhang X., Kainz V., Zhao J., Strassman A. M., Levy D. (2013). Vascular extracellular signal-regulated kinase mediates migraine-related sensitization of meningeal nociceptors. *Annals of Neurology*.

[66] Hoskin K. L., Bulmer D. C. E., Goadsby P. J. (1999). Fos expression in the trigeminocervical complex of the cat after stimulation of the superior sagittal sinus is reduced by L-NAME. *Neuroscience Letters*.

[67] Helyes Z., Pozsgai G., Börzsei R. (2007). Inhibitory effect of PACAP-38 on acute neurogenic and non-neurogenic inflammatory processes in the rat. *Peptides*.

[68] Wu H.-G., Jiang B., Zhou E.-H. (2008). Regulatory mechanism of electroacupuncture in irritable bowel syndrome: preventing MC activation and decreasing SP VIP secretion. *Digestive Diseases and Sciences*.

[69] Qiao L.-N., Liu J.-L., Tan L.-H., Yang H.-L., Zhai X., Yang Y.-S. (2017). Effect of electroacupuncture on thermal pain threshold and expression of calcitonin-gene related peptide, substance P and *γ*-aminobutyric acid in the cervical dorsal root ganglion of rats with incisional neck pain. *Acupuncture in Medicine*.

[70] Guo H., Zhu S.-F., Zhang R.-R., Zhao X.-L., Wan M.-H., Tang W.-F. (2014). Electroacupuncture ameliorates acute lung injury through promoting gastrointestinal motility in rats with acute pancreatitis. *Evidence-Based Complementary and Alternative Medicine*.

[71] Zhu J., Chen X.-Y., Li L.-B. (2015). Electroacupuncture attenuates collagen-induced arthritis in rats through vasoactive intestinal peptide signalling-dependent re-establishment of the regulatory T cell/T-helper 17 cell balance. *Acupuncture in Medicine*.

[72] Lu Z.-Z., Yin X.-J., Teng W.-J. (2015). Comparative effect of electroacupuncture and moxibustion on the expression of substance P and vasoactive intestinal peptide in patients with irritable bowel syndrome. *Journal of Traditional Chinese Medicine*.

[73] Eshkevari L., Egan R., Phillips D. (2012). Acupuncture at ST36 prevents chronic stress-induced increases in neuropeptide Y in rat. *Experimental Biology and Medicine*.

[74] Zhang L., Huang Z., Shi X. (2018). Protective effect of electroacupuncture at zusanli on myocardial injury in septic rats. *Evidence-Based Complementary and Alternative Medicine*.

[75] Song X.-J., Zhang D., Wang S.-Y., Li S.-Y. (2014). Investigation of hepatic blood perfusion by laser speckle imaging and changes of hepatic vasoactive substances in mice after electroacupuncture. *Evidence-Based Complementary and Alternative Medicine*.

[76] Yang J., Zeng F., Feng Y. (2012). A PET-CT study on the specificity of acupoints through acupuncture treatment in migraine patients. *BMC Complementary and Alternative Medicine*.

[77] Gao Z., Liu X., Yu S. (2014). Electroacupuncture at acupoints reverses plasma glutamate, lipid, and LDL/VLDL in an acute migraine rat model: a ^1^H NMR-based metabolomic study. *Evidence-Based Complementary and Alternative Medicine*.

[78] Zhang H., He S.-D., Hu Y.-P., Zheng H. (2016). Antagonism of cannabinoid receptor 1 attenuates the anti-inflammatory effects of electroacupuncture in a rodent model of migraine. *Acupuncture in Medicine*.

[79] Xue T., Yuan K., Cheng P. (2013). Alterations of regional spontaneous neuronal activity and corresponding brain circuit changes during resting state in migraine without aura. *NMR in Biomedicine*.

[80] Jia Z., Chen X., Tang W., Zhao D., Yu S. (2019). Atypical functional connectivity between the anterior cingulate cortex and other brain regions in a rat model of recurrent headache. *Molecular Pain*.

[81] Maleki N., Becerra L., Brawn J., McEwen B., Burstein R., Borsook D. (2013). Common hippocampal structural and functional changes in migraine. *Brain Structure & Function*.

[82] Li Z., Liu M., Lan L. (2016). Altered periaqueductal gray resting state functional connectivity in migraine and the modulation effect of treatment. *Scientific Reports*.

