# Erratum to “Effect of Electroacupuncture on Hyperalgesia and Vasoactive Neurotransmitters in a Rat Model of Conscious Recurrent Migraine”

**DOI:** 10.1155/2019/8181245

**Published:** 2019-07-14

**Authors:** Xiaobai Xu, Lu Liu, Luopeng Zhao, Bin Li, Xianghong Jing, Zhengyang Qu, Yupu Zhu, Yajie Zhang, Zhijuan Li, Marc Fisher, Brian E. Cairns, Linpeng Wang

**Affiliations:** ^1^Acupuncture and Moxibustion Department, Beijing Hospital of Traditional Chinese Medicine, Capital Medical University, Beijing Key Laboratory of Acupuncture Neuromodulation, Beijing, China; ^2^Beijing Hospital of Traditional Chinese Medicine, Capital Medical University, Beijing Institute of Traditional Chinese Medicine, Beijing, China; ^3^Institute of Acupuncture and Moxibustion, China Academy of Chinese Medical Sciences, Beijing, China; ^4^Division of Stroke and Cerebrovascular Diseases, Department of Neurology, Beth Israel Deaconess Medical Center, Boston, MA, USA; ^5^Faculty of Pharmaceutical Sciences, The University of British Columbia, Vancouver, Canada; ^6^Center for Neuroplasticity and Pain, SMI, Department of Health Science and Technology, The Faculty of Medicine, Aalborg University, Aalborg E, Denmark

In the article titled “Effect of Electroacupuncture on Hyperalgesia and Vasoactive Neurotransmitters in a Rat Model of Conscious Recurrent Migraine” [1], the name of the eleventh author was given incorrectly as Brain E. Cairns. The author's name should have been written as Brian E. Cairns. The revised authors' list is shown above and the Authors' Contributions section should be updated as follows:

Linpeng Wang, Bin Li, Xianghong Jing, Marc Fisher, and Brian E. Cairns designed the study; Luopeng Zhao, Zhengyang Qu, and Yupu Zhu carried out the animal experiments; Yajie Zhang and Zhijuan Li coordinated the experiments; Xiaobai Xu and Lu Liu participated in data analyses. All authors read and approved the final manuscript.
